# Fully biodegradable versus nitinol occluder devices for transcatheter patent foramen ovale closure: a systematic review

**DOI:** 10.21542/gcsp.2026.34

**Published:** 2026-08-31

**Authors:** Abdulbari Alsaeed, Ahmad R. Al-Qudimat, Mohammed Qintar, Raed M. Al-Zoubi, Rufaidah H. Alameen, Osama Mohamad

**Affiliations:** 1 Surgical Research Section, Department of Surgery, Hamad Medical Corporation, Doha, Qatar; 2 QU Health, Qatar University, Doha, Qatar; 3 Department of Cardiology, Heart Hospital, Hamad Medical Corporation, Doha, Qatar; 4 Medical Education Department, Hamad Medical Corporation, Doha, Qatar; 5 School of Medicine, Istanbul Medipol University, Istanbul, Turkey

## Abstract

**Background:** The optimal device platform for transcatheter patent foramen ovale (PFO) closure remains uncertain. Permanent nitinol occluders are widely used, but concerns persist regarding long-term foreign-body exposure and device-related complications. Fully biodegradable occluders have been developed to provide temporary septal support during endothelialization before gradual resorption, with the intended benefit accruing only after the device has resorbed.

**Methods:** We systematically searched Medline, PubMed, Embase, Scopus, Web of Science, the Cochrane Library, and trial registries through 17 February 2026. Eligible studies were randomized or comparative observational studies of patients aged 16 years or older undergoing PFO closure with biodegradable or bioresorbable versus nitinol or metallic occluders. Two reviewers independently screened studies and extracted data. Risk of bias was assessed using RoB 2 for randomized evidence and ROBINS-I for non-randomized evidence. Because of clinical and methodological heterogeneity, findings were synthesized narratively.

**Results:** Three comparative studies were included—one randomized trial and two retrospective cohorts—comprising 481 analyzed participants. Procedural success was high with both device types. Residual shunt generally decreased over time, with no clear advantage for either platform. Serious adverse events, device thrombosis, embolization or detachment, and atrial arrhythmias were uncommon, though event counts were small. Two of the three studies were migraine-specific cohorts, and the randomized trial enrolled a mixed-indication population in which treatment-resistant migraine was the most common single indication; the available evidence therefore reflects device-platform performance predominantly in migraine populations. No study followed participants substantially beyond the expected biodegradation window.

**Conclusion:** Current evidence suggests similar short- to mid-term performance of biodegradable and nitinol occluders, but certainty is low to very low, and no study yet assesses the post-resorption period in which any advantage of a biodegradable platform would be expected to emerge. Larger multicenter studies with standardized imaging, rhythm monitoring, and follow-up extending beyond biodegradation are needed. Because two of the three studies were migraine-specific and event counts were small, these findings should not be extrapolated to stroke-prevention populations, and the absence of observed between-device differences should not be interpreted as equivalence.

## Introduction

Patent foramen ovale (PFO) is a remnant of the fetal circulation that persists when the septum primum and septum secundum fail to fuse after birth. Epidemiological studies indicate that a PFO is present in approximately 25% of adults^1,2^. Although usually incidental and asymptomatic, a PFO may permit intermittent right-to-left shunting during transient increases in right atrial pressure, allowing paradoxical embolism. This mechanism has been strongly associated with cryptogenic ischemic stroke, particularly in younger patients without conventional vascular risk factors^3^.

The global burden of stroke remains substantial. According to the Global Burden of Disease 2019 Study, stroke is the second leading cause of death and a major cause of long-term disability worldwide, accounting for more than 12 million incident cases annually and contributing substantially to disability-adjusted life years (DALYs)^4^. Cryptogenic stroke constitutes approximately 25–30% of ischemic strokes, and a PFO is identified in up to 40–50% of these cases^5^. Given the high background prevalence of PFO, distinguishing an incidental PFO from a causally related one is essential. Risk-stratification tools such as the Risk of Paradoxical Embolism (RoPE) score estimate the probability that a PFO is stroke-related and help guide treatment decisions^6,7^.

Transcatheter PFO closure has become a standard strategy for secondary stroke prevention in carefully selected patients, supported by randomized controlled trials showing reduced recurrent ischemic stroke compared with antiplatelet therapy alone. The REDUCE trial, for example, found that device closure plus antiplatelet therapy significantly lowered the risk of recurrent ischemic stroke, though at the cost of a higher incidence of early atrial arrhythmias^8^. Current practice relies primarily on permanent nitinol double-disc occluders, which provide durable mechanical sealing but remain lifelong implants. Although efficacy is favorable, residual shunting or incomplete closure may be seen on follow-up imaging, potentially compromising long-term protection and prompting further investigation or re-intervention in selected cases^9^. In addition, even relatively infrequent device-related adverse events—including atrial fibrillation, thrombus formation, and other device-associated complications—matter when comparing platforms and informing shared decision-making^8,9^.

Biodegradable, or bioresorbable, PFO occluders have been developed to provide temporary septal support during endothelialization, followed by gradual resorption to reduce long-term foreign-body exposure. Despite this theoretical advantage, the comparative effectiveness and safety of biodegradable devices relative to conventional nitinol occluders remain unclear. The available studies are few, often observational, and heterogeneous in patient selection, definitions of complete closure and residual shunt, duration of follow-up, and adverse-event reporting. No prior systematic review has comprehensively synthesized outcomes between fully biodegradable or bioresorbable and permanent nitinol occluders—a gap this review sets out to address. We therefore aimed to evaluate and compare the efficacy and safety of fully biodegradable or bioresorbable versus nitinol occluders for transcatheter PFO closure.

Because the available comparative studies include both mixed-indication and migraine-specific populations, this review evaluates biodegradable versus nitinol devices as platforms, rather than drawing indication-specific conclusions for stroke prevention or migraine management.

## Methods

This systematic review was conducted and reported in accordance with the Preferred Reporting Items for Systematic Reviews and Meta-Analyses (PRISMA) guidelines^10^. The protocol was registered in PROSPERO before data extraction (registration number CRD420261305358)^11^.

### Study eligibility and selection

Using the PICOS framework, we included randomized controlled trials and comparative observational studies (e.g., cohort or registry studies) of patients aged ≥16 years undergoing transcatheter PFO closure in which a fully biodegradable or bioresorbable device was directly compared with a permanent nitinol occluder.

We excluded non-comparative studies (case reports or series without a concurrent comparator); reviews, editorials, or letters without extractable comparative data; animal or in-vitro studies; and conference abstracts with insufficient data. Studies focusing primarily on atrial septal defect (ASD) or other congenital heart defects were excluded; mixed PFO and ASD cohorts were included only when PFO-specific outcomes were extractable.

### Outcome measures

Primary outcomes were: (1) residual right-to-left shunt on follow-up contrast echocardiography, reported as any residual shunt and/or moderate-to-large residual shunt according to each study’s definition; (2) serious adverse events (SAEs) as defined by the original study; and (3) new-onset atrial fibrillation or flutter (AF/AFL) during follow-up. Secondary outcomes were procedural success, device thrombosis, embolization/malposition/detachment, reintervention (repeat closure or surgical removal), all-cause mortality, neurologic events (recurrent stroke or transient ischemic attack [TIA]), and migraine-related outcomes (e.g., Migraine Disability Assessment [MIDAS] score, attack frequency, responder rate), where reported.

### Literature search strategy

We searched MEDLINE (*via* EBSCOhost), PubMed, Embase (Elsevier), Cochrane CENTRAL (*via* the Cochrane Library), Scopus, and Web of Science. The final search was run on 17 February 2026. Search strategies combined controlled-vocabulary and free-text terms for PFO, transcatheter or percutaneous closure, occluder devices, biodegradable, bioresorbable, or bioabsorbable materials, and comparator terms such as nitinol occluder. We also searched the World Health Organization International Clinical Trials Registry Platform (ICTRP) and ClinicalTrials.gov. No language restrictions were applied. Duplicate records were removed with Rayyan (Rayyan Systems Inc.). Full search strategies for each database are provided in the Supplement.

### Study selection

Two reviewers independently screened titles and abstracts in Rayyan and then assessed full texts against the eligibility criteria. Disagreements were resolved by discussion, with a third reviewer adjudicating when needed.

### Data collection

Two reviewers independently extracted data using a piloted, standardized spreadsheet form. Extracted items included study characteristics (year, country, design, setting, recruitment period, funding, conflicts of interest); participant characteristics (age, sex, indication, baseline shunt grade, atrial septal aneurysm, tunnel length, and other anatomical features when reported); device characteristics (brand or model, material, size); procedural guidance (transesophageal, intracardiac, or transthoracic echocardiography [TEE/ICE/TTE]); anesthesia and technical approach; antithrombotic regimen (peri- and post-procedural drugs and duration); and outcomes at each reported time point, with definitions and methods of ascertainment.

### Risk-of-bias assessment

For randomized controlled trials, the Cochrane Risk of Bias tool, version 2 (RoB 2)^12^, was used to evaluate risk of bias at the outcome level. RoB 2 assesses five domains: (1) the randomization process; (2) deviations from intended interventions; (3) missing outcome data; (4) measurement of the outcome; and (5) selection of the reported result. Each domain was rated as low risk, some concerns, or high risk of bias, yielding an overall judgment for each trial.

Risk of bias in non-randomized comparative studies was assessed with the Risk Of Bias In Non-randomized Studies of Interventions tool (ROBINS-I)^13^. ROBINS-I evaluates seven domains: confounding, selection of participants, classification of interventions, deviations from intended interventions, missing data, measurement of outcomes, and selection of the reported result. Each domain was judged as low, moderate, serious, or critical risk, yielding an overall judgment for each study. The confounding domains considered clinically relevant were baseline PFO anatomy, shunt severity, migraine severity, indication for closure, device-selection factors, operator experience, and differences in follow-up or outcome ascertainment.

The overall certainty of evidence for the main outcomes was summarized using a GRADE-informed approach and is presented in the Supplement. Certainty was assessed by outcome, considering risk of bias, inconsistency, indirectness, imprecision, and other considerations. Because meta-analysis was not performed, certainty judgments were based on study design, risk of bias, consistency of findings across studies, outcome-definition heterogeneity, event counts, and clinical directness.

## Results

### Literature review

**Figure 1.**
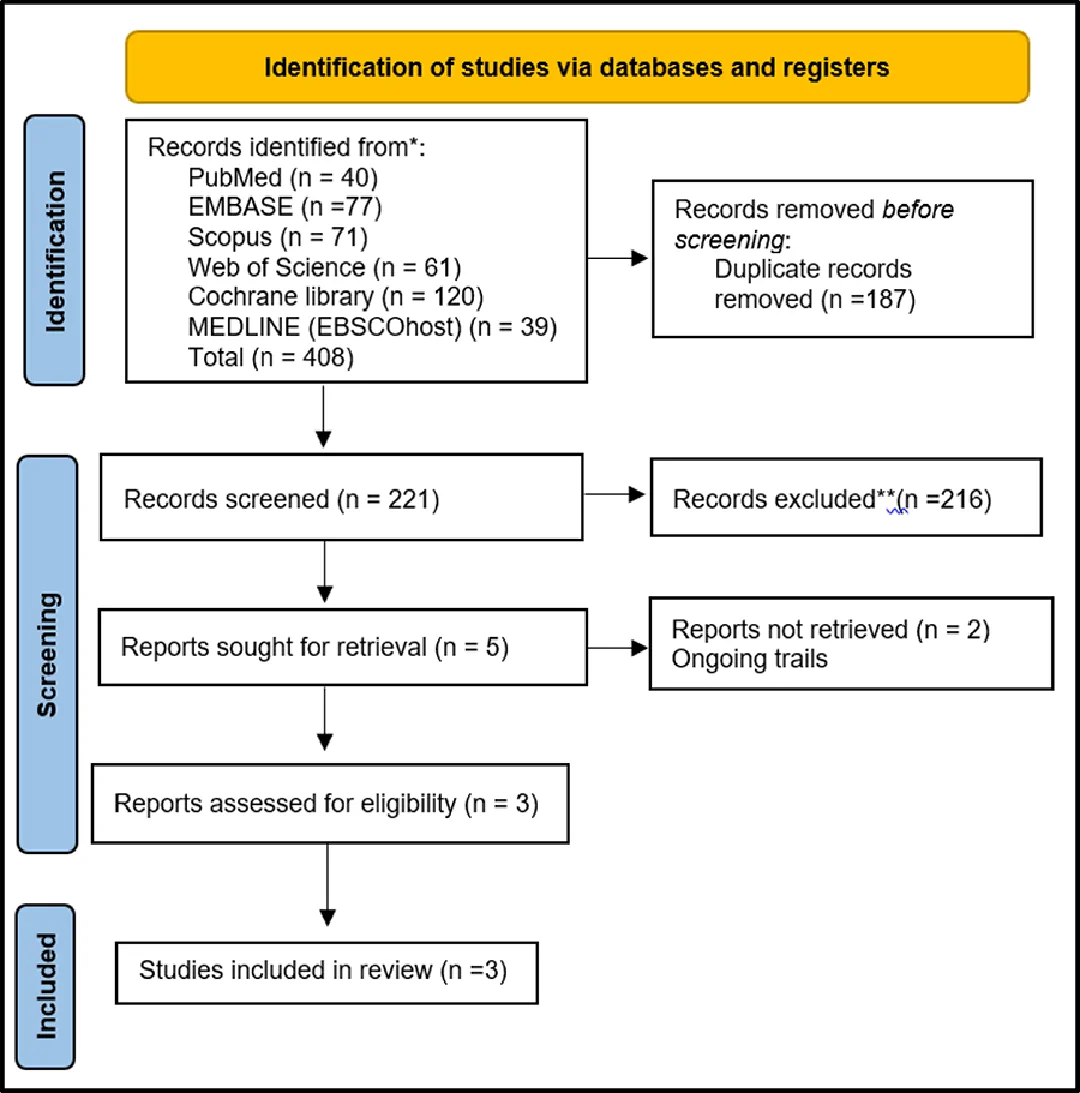
PRISMA 2020 flow diagram of study selection.

The search identified 408 records. After 187 duplicates were removed, 221 records underwent title and abstract screening, of which 216 were excluded. Five reports were sought for full-text assessment; two could not be retrieved because the relevant trial reports were not yet published in full. The remaining three studies^14–16^ were assessed against the eligibility criteria and all met them (Figure 1). The two unretrieved reports corresponded to registered but not-yet-reported randomized trials: the BioMetal trial (ClinicalTrials.gov NCT06203873) and the BIOCLOSE-PFO trial (NCT07300358).

**Figure 2.**
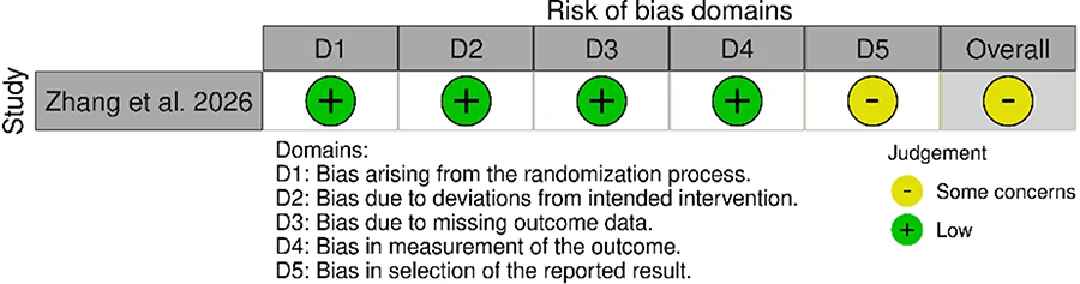
Risk of bias assessment of the randomized controlled trial using RoB 2.

**Figure 3.**
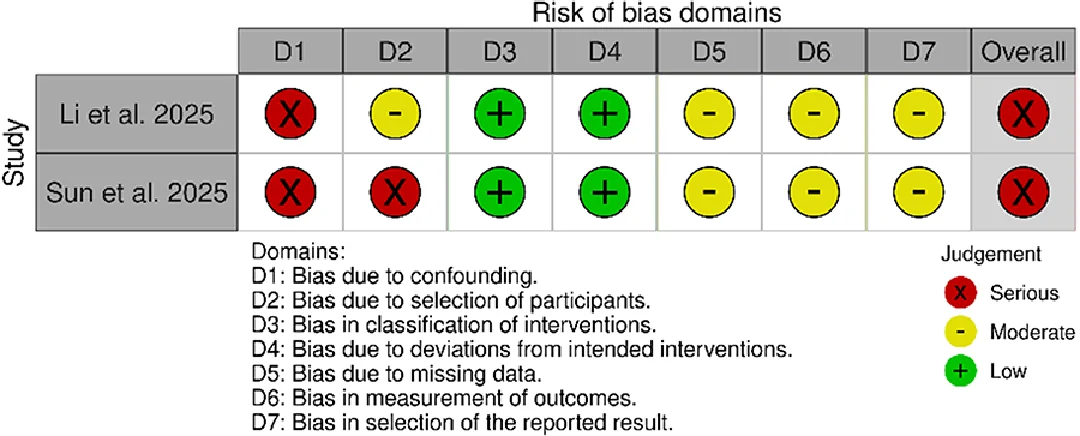
Risk of bias assessment of non-randomized comparative studies using ROBINS-I.

### Risk of bias assessment

The randomized controlled trial by Zhang et al. was assessed using RoB 2 (Figure 2). The overall judgment was *some concerns*, driven mainly by Domain 5 (selection of the reported result), as a fully prespecified statistical analysis plan was not clearly available. Domains 1–4 were judged at low risk of bias, reflecting the reported randomization process, the absence of major deviations from intended interventions, low missing outcome data, and no likely systematic bias in outcome measurement.

The two retrospective cohorts were assessed using ROBINS-I, and both were judged at serious risk of bias overall (Figure 3). For Li et al., this reflected potential confounding by indication, device-selection factors, baseline clinical and anatomical differences, and the retrospective single-centre design. For Sun et al., it reflected potential confounding, selection of participants into the final analyzed cohort, and incomplete standardization of follow-up and outcome ascertainment. Neither was judged at critical risk, so both were retained in the synthesis. Domain-level RoB 2 and ROBINS-I judgments are provided in Tables S1 and S2.

### Study characteristics

Three studies, all conducted in China, were included: one multicentre randomised trial and two single-centre retrospective cohorts. In total, 493 participants were enrolled and 481 were analysed (biodegradable *n* = 233; nitinol *n* = 248). The randomised trial enrolled patients undergoing PFO closure for mixed indications; both cohorts enrolled patients with migraine and PFO. Mean age across studies ranged from the late 30s to the early 50s. Female participants predominated in all three studies, comprising 70.1% of the analysed population (337/481). Device specifications and peri- and post-procedural antithrombotic regimens varied across studies. Study characteristics are summarised in Table 1.

**Table 1:** Characteristics of included studies.

Characteristic	**Zhang et al.**	**Li et al.**	**Sun et al.**
Year	2026	2025	2025
Country	China	China	China
Design	RCT	Retrospective	Retrospective
Setting	Multicenter	Single centre	Single centre
Recruitment years	Feb–Jul 2021	Oct 2023–Jan 2024	Jan 2021–Jun 2024
Sample size enrolled (total)	190	158	145
Participant demographics			
Analyzed sample size, biodegradable group	96	81	56
Sample size analysed Nitinol	94	77	77
Indication	Mixed indications (including cryptogenic stroke/TIA; …)	Migraine	Migraine
Age (mean±SD) Bio	40.95 ± 11.70	45.58 ± 13.93	36.84 ± 11.01
Age (mean±SD) Nitinol	43.87 ± 10.78	51.55 ± 13.85	40.90 ± 13.01
Sex (M/F) Bio	26/70	21/60	18/38
Sex (M/F) Nitinol	32/62	29/48	18/59
Device specifications			
Biodegradable device brand/model	Novel biodegradable PFO occluder (trial device)	MemoSorb^®^ PFO occluder	NR
Biodegradable material	PDO monofilament + PLLA membranes	PLA-based framework; PDO monofilament; PLLA membranes	NR
Comparator device brand/model	Cardi-O-fix PFO occluder	Nitinol occluders (Amplatzer™ + Shanghai oxidized membrane single-rivet types)	Shanghai Shape Memory Alloy LTD or Beijing Huayi Shengjie LTD
Comparator material	Nitinol	Nitinol	Nitinol
Imaging guidance (TEE/ICE/TTE)	Trial: TTE-guided; Control: fluoroscopy-guided	Intraprocedural: ICE; morphology: TEE; follow-up: cTTE bubble	Follow-up: cTTE at 1, 6, 12 months (intraprocedural NR)
Anticoagulation/antiplatelet therapy			
Peri-procedure anticoag/antiplatelet	Heparin 48 h	No antiplatelet loading dose administered	LMWH 48 h
Post-procedure regimen	Aspirin 100 mg/day + clopidogrel 50–75 mg/day	DAPT aspirin 100 mg + clopidogrel 75 mg daily, then SAPT	Aspirin 6 months + clopidogrel 3 months
DAPT duration	6 months	6 months^†^	3 months (aspirin+clopidogrel)
SAPT duration	NR	6 months	3 months (aspirin only)
Anticoagulation duration	Heparin 48 h	NR	LMWH 48 h

**Notes.**

Bio biodegradable PLLA Poly-L-lactic acid PDO Polydioxanone DAPT dual antiplatelet therapy SAPT single antiplatelet therapy ICE intracardiac echo TEE transesophageal echo TTE/cTTE (contrast) transthoracic echo NR not reported

† Total antiplatelet duration: 12 months (6m DAPT + 6m SAPT).

The difference between the 493 enrolled and 481 analysed participants arose entirely in Sun et al., in which 145 patients were enrolled before the procedure and 12 did not contribute to the analysed cohort (biodegradable group: 1 declined the procedure and 3 were lost to follow-up; metallic group: 2 had intraprocedural pericardial tamponade, 1 procedure was aborted for failed guidewire crossing, and 5 were lost to follow-up), leaving 133 analysed. In Zhang et al., all 190 randomised patients were analysed under intention-to-treat; in Li et al., 158 patients were analysed (168 screened → 164 allocated → 158 analysed). A per-study flow from enrolment to implantation to evaluable imaging at each follow-up time point is provided in Table S5.

### Baseline shunt severity and anatomy

In all studies, most participants had moderate-to-large baseline right-to-left shunts, and small shunts were uncommon. Zhang et al. reported small/moderate/large shunt grades of 0/27/69 versus 3/28/63 in the biodegradable and nitinol groups, respectively; Li et al. reported 0/33/48 versus 0/28/49; and Sun et al. reported 0/16/40 versus 0/34/43. Atrial septal aneurysm (ASA) was rare in Zhang et al. (3/96 vs 4/94) and more common in Li et al. (12/81 vs 11/77); Sun et al. did not report ASA. PFO tunnel length was reported only by Li et al. (biodegradable 8.71 ± 3.78 mm versus nitinol 8.55 ± 3.96 mm). Baseline shunt severity and key anatomical features are summarised in Table 2.

**Table 2:** Baseline shunt severity and anatomical characteristics.

	**Zhang et al.**	**Li et al.**	**Sun et al.**
Baseline shunt grade scale used	Contrast echo: RLS grade 1–3 (Valsalva)	cTTE bubble: RLS grade II–III (Valsalva)	cTEE/cTTE bubble: Grade II vs Grade III+
Baseline shunt grade distribution, nGrade I/Grade II/Grade III+ Bio	0/27/69	0/33/48	0/16/40
Baseline shunt grade distribution, nGrade I/Grade II/Grade III+ Nitinol	3/28/63	0/28/49	0/34/43
ASA Bio n/N (%)	3/96 (3.13%)	12/81 (14.81%)	NR
ASA Nitinol n/N (%)	4/94 (4.26%)	11/77 (14.29%)	NR
PFO tunnel length Bio	NR	8.71 ± 3.78 mm	NR
PFO tunnel length Nitinol	NR	8.55 ± 3.96 mm	NR

**Notes.**

ASA atrial septal aneurysm Bio biodegradable NR not reported

Sun et al. included only patients with grade II or higher right-to-left shunt at baseline; therefore, grade I/small shunt was 0 in both groups.

### Efficacy outcome

#### Residual shunt

In the multicentre randomised trial by Zhang et al., the primary efficacy endpoint was PFO closure success at 6 months, assessed by contrast echocardiography. Successful closure was achieved in 87/96 patients (90.6%) in the biodegradable group and 86/94 (91.5%) in the nitinol group, meeting the predefined non-inferiority criterion. Moderate-to-large residual right-to-left shunt at 6 months occurred in 9/96 and 8/94 patients, respectively. At 24 months, it was reported in 5/96 and 8/94 patients, with no statistically significant between-group difference. These findings suggest that closure efficacy was maintained through the degradation period, although the study was not powered to detect small differences in residual shunt at later follow-up.

Li et al. reported moderate-to-large residual shunt in 28/74 versus 27/71 patients at 6 months and in 15/71 versus 12/68 at 12 months, in the biodegradable and nitinol groups respectively. In Sun et al., moderate-to-large residual shunt declined over follow-up—from 1 month (4/52 vs 8/70) to 6 months (3/45 vs 7/63) to 12 months (1/38 vs 4/51)—in the biodegradable and metallic groups respectively. Moderate-to-large residual shunt by follow-up time point is summarised in Table 3A.

**Table 3A:** Moderate-to-large residual right-to-left shunt by follow-up time point. Moderate-to-large residual shunt refers to grade 2 or 3 residual RLS according to the original study definition.

**Study**	**Time point**	**Biodegradable n/N (%)**	**Nitinol/metallic n/N (%)**
Zhang et al.	6 months	9/96 (9.4%)	8/94 (8.5%)
Zhang et al.	24 months	5/96 (5.2%)	8/94 (8.5%)
Li et al.	6 months	28/74 (37.8%)	27/71 (38.0%)
Li et al.	12 months	15/71 (21.1%)	12/68 (17.6%)
Sun et al.	1 month	4/52 (7.7%)	8/70 (11.4%)
Sun et al.	6 months	3/45 (6.7%)	7/63 (11.1%)
Sun et al.	12 months	1/38 (2.6%)	4/51 (7.8%)

Any residual shunt was reported only in the cohort studies. In Li et al., it was common at 6 months (57/74 vs 55/71) and decreased by 12 months (30/71 vs 24/68) in the biodegradable and nitinol groups respectively. In Sun et al., it decreased over time—from 12/52 versus 21/70 at 1 month, to 11/45 versus 17/63 at 6 months, to 8/38 versus 12/51 at 12 months. Any residual shunt is summarised in Table 3B.

**Table 3B:** Any residual right-to-left shunt by follow-up time point. Any residual shunt includes mild, moderate, or large residual RLS, according to each study’s reporting.

**Study**	**Time point**	**Biodegradable n/N (%)**	**Nitinol/metallic n/N (%)**
Li et al.	6 months	57/74 (77.0%)	55/71 (77.5%)
Li et al.	12 months	30/71 (42.3%)	24/68 (35.3%)
Sun et al.	1 month	12/52 (23.1%)	21/70 (30.0%)
Sun et al.	6 months	11/45 (24.4%)	17/63 (27.0%)
Sun et al.	12 months	8/38 (21.1%)	12/51 (23.5%)

Overall, residual shunt decreased over time in both the biodegradable and the nitinol/metallic groups, and no study showed a clear residual-shunt advantage for either device type. Interpretation is limited by heterogeneity in shunt definitions, imaging protocols, follow-up intervals, and denominators.

Because contrast-echocardiographic follow-up was incomplete in the cohort studies, the residual-shunt denominators at each time point reflect only patients with follow-up imaging rather than the full analysed cohort (Li et al.: biodegradable 74 then 71, nitinol 71 then 68, at 6 and 12 months; Sun et al.: biodegradable 52, 45, and 38, metallic 70, 63, and 51, at 1, 6, and 12 months), whereas Zhang et al. analysed all patients under intention-to-treat, giving stable denominators of 96 and 94. The proportion without follow-up imaging at the final time point was similar between device groups within each cohort (Li et al. 12.3% vs 11.7%; Sun et al. 32.1% vs 33.8%), so the reduction in residual shunt over time is unlikely to be explained by differential attrition between device types. Nonetheless, the substantial absolute loss in Sun et al. means its later estimates should be read as imaging-subset rather than intention-to-treat results.

At the nearest common time point (6 months), moderate-to-large residual shunt was similar between device groups within every study (Zhang et al. 9.4% vs 8.5%; Li et al. 37.8% vs 38.0%; Sun et al. 6.7% vs 11.1%). The large *between-study* difference in absolute rates—approximately 7–11% in Zhang and Sun versus approximately 38% in Li—reflects differing residual-shunt definitions and imaging modalities (Table S6) rather than device performance. Given this heterogeneity, a quantitative meta-analysis was not undertaken; narrative synthesis was pre-specified in the registered protocol (PROSPERO CRD420261305358). The direction of effect was nonetheless consistent, with neither device type showing a clear residual-shunt advantage at any comparable time point.

#### Procedural success

For this review, procedural (technical) success was defined as successful device deployment and release in a stable position at the end of the index procedure, without device removal, embolisation, or surgical conversion. The underlying denominators differed across studies: Zhang et al. reported success against the randomised population (intention-to-treat), Li et al. within the analysed cohort, and Sun et al. only among patients who were both successfully implanted and completed follow-up—so the Sun figures do not represent a strict intention-to-treat procedural-success rate.

Procedural success was high across all studies, supporting the technical feasibility of biodegradable PFO occluder implantation in experienced centres. In Zhang et al., success was achieved in 95/96 patients in the biodegradable group and 94/94 in the nitinol group. In Li et al., it was 100% in both groups (81/81 biodegradable; 77/77 nitinol). In Sun et al., 56/56 biodegradable and 77/77 metallic patients were successfully implanted—but, as noted, these reflect the final analysed cohort rather than the procedural denominator and should be read as successful implantation among included follow-up patients.

Overall, the available data suggest high procedural feasibility for both biodegradable and nitinol/metallic occluders, though procedural-success definitions and denominators were not fully consistent across studies.

### Safety outcomes

#### Serious adverse events

Serious adverse events (SAEs) and major device-related complications were rare across the three studies. In the randomised trial, one SAE occurred in the biodegradable group (1/96), requiring surgical removal of the device, and one in the nitinol group (1/94), involving femoral vein puncture-site bleeding. Neither retrospective cohort reported any SAE (Li et al. 0/81 vs 0/77; Sun et al. 0/56 vs 0/77).

#### Thrombosis, embolization/malposition/detachment, reintervention

Device thrombosis was not observed in any study (all groups, zero events). Embolisation or malposition events were also uncommon: Li et al. reported one occluder detachment in the biodegradable arm (1/81) and none in the nitinol arm (0/77), while Zhang et al. and Sun et al. reported no such events in either group.

#### Atrial fibrillation/arrhythmia

Atrial fibrillation or flutter (AF/AFL) was uncommon across the three studies. In the randomised trial, no AF/AFL was reported in either group during early follow-up (≤30 days) or through 6 months (0/96 vs 0/94). Other arrhythmias (e.g., sinus arrhythmia, sinus bradycardia) occurred at similar rates in the two groups (5/96 vs 6/94 at 1 month) but resolved without intervention. In Li et al., transient AF after implantation was noted in 3/81 patients in the biodegradable group and 2/77 in the nitinol group. In Sun et al., no AF/AFL was observed in either arm (0/56 vs 0/77), on short-term ECG monitoring.

#### Other peri-procedural events

Sun et al. described minor peri-procedural events (including transient atrioventricular block, small pericardial effusion, fever, and hypotension) without progression to serious complications.

#### Mortality and stroke/TIA recurrence

Mortality and recurrent neurological events were infrequent and inconsistently reported. In the randomised trial, no deaths occurred in either group (0/96 biodegradable vs 0/94 nitinol). Stroke or TIA recurrence was reported only by Li et al., where no events were observed at 12 months in either group (0/81 vs 0/77). Sun et al. did not report extractable data on stroke or TIA recurrence, limiting comparative inference.

#### Migraine outcomes

Migraine outcomes were reported in the two retrospective migraine-specific cohorts, though not in a fully comparable format. In Sun et al., both device groups improved postoperatively in MIDAS score and monthly migraine attack days, with no statistically significant difference between biodegradable and metallic occluders at 12 months: postoperative MIDAS score was 10.45 ± 9.19 versus 11.32 ± 9.62 (*p* = 0.453), and monthly migraine attack days were 2.09 ± 1.58 versus 1.87 ± 1.43 (*p* = 0.506). Although Sun et al. defined migraine improvement as complete symptom elimination or a >50% reduction in monthly attacks, responder-rate data were not reported in a format allowing direct comparison with Li et al.

Li et al. likewise reported significant postoperative improvement in both device groups, with no significant between-device difference in migraine relief on Kaplan–Meier analysis (*p* = 0.644). Overall, the available migraine-specific evidence suggests comparable short-term symptom improvement after biodegradable and nitinol/metallic PFO closure, but certainty is limited: both studies were retrospective and outcome reporting was inconsistent.

### Certainty of evidence

The certainty of evidence was low to very low across the main outcomes. The principal reasons for downgrading were the small number of included studies, the predominance of retrospective observational evidence, limited sample sizes, inconsistent outcome definitions, variable follow-up intervals, and imprecision from rare safety events. Certainty was highest for short-term procedural feasibility and moderate-to-large residual shunt, as these were reported across all three studies and informed by the one randomised trial—though it remained limited because outcome definitions, imaging protocols, and denominators differed. Certainty was very low for rare adverse events—serious adverse events, AF/AFL, device thrombosis, embolisation or detachment, and mortality—because event counts were very small and surveillance methods inconsistent. It was likewise very low for migraine outcomes, which came only from two retrospective migraine-specific cohorts with incompletely comparable reporting.

For the two outcomes of greatest clinical interest, this certainty carries a specific interpretation. For moderate-to-large residual shunt (low certainty) and serious adverse events (very low certainty), the absence of a demonstrable between-device difference should not be read as evidence of equivalence: event counts were small and the corresponding confidence intervals wide, so clinically important differences in either direction cannot be excluded. These findings are hypothesis-generating and do not establish non-inferiority of either platform for safety.

## Discussion

This systematic review included three comparative studies—one multicentre RCT and two retrospective cohorts, all conducted in China—with 481 analysed participants (biodegradable *n* = 233; nitinol *n* = 248). Procedural success was consistently high in both device groups, suggesting that biodegradable occluder implantation is technically feasible in experienced centres. Certainty of evidence, however, was low to very low across outcomes, reflecting the small evidence base, the observational design of two of the three studies, inconsistent outcome definitions, and imprecision for rare adverse events.

Residual shunting decreased over time in both device groups, with similar rates of moderate-to-large residual shunt at comparable follow-up points. This pattern aligns with broader post-closure experience using nitinol devices, in which incomplete or persistent right-to-left shunting is common early after implantation but tends to lessen over time, likely through progressive endothelialisation and sealing^17^. In one large nitinol-closure cohort, for example, right-to-left shunting was seen in 19.5% of patients at around 4 months, falling to 8.4% by approximately 11 months, with a small proportion showing persistent mild positivity at 2 years^9^. These external data make our findings biologically plausible and consistent with time-dependent sealing, while also underlining that “any residual shunt” depends heavily on how shunt is defined and measured^18^. Residual shunt is commonly graded by the number of bubbles detected on transoesophageal echocardiography (TEE) or transcranial Doppler (TCD) during a Valsalva manoeuvre—successful closure <10 bubbles, moderate/large ≥10 bubbles^19,20^—a threshold that varied across the included studies.

In our review, serious device-related complications were rare, and short-term arrhythmias and thrombosis were uncommon in both device groups. In the randomised trial, no deaths, embolism, device thrombus, or erosion were observed over 24 months, supporting short-term procedural safety under trial conditions^14,17^. An important rationale for biodegradable platforms, however, is not early safety alone but the aim of reducing the lifelong foreign-body burden of nitinol. Nitinol devices are widely used, yet their non-degradable nature has raised concerns about potential long-term complications—arrhythmias, thromboembolic events, and infection—and about the practicality of future procedures requiring transseptal access, such as left-sided ablation or left atrial appendage intervention^21–23^. Consistent with this concern, the biomaterials and device-design literature describes complications including malposition or embolisation, erosion, and thrombus formation; although uncommon, some later events can be severe enough to require surgical removal, motivating the development of fully degradable devices^24^.

A further long-term dimension is material hypersensitivity. In a randomised trial of PFO closure in patients with nickel hypersensitivity, symptom-based “device syndrome” occurred substantially more often in nickel-hypersensitive patients^25^. Although the studies we included did not centre on nickel allergy, this literature strengthens the clinical rationale for biodegradable devices that may reduce prolonged nickel exposure.

The included studies reported very low mortality and limited extractable neurological-recurrence data, so strong between-device conclusions for stroke prevention cannot be drawn. The broader randomised evidence base, in cryptogenic-stroke populations with high-risk PFO features, indicates that closure reduces recurrent stroke compared with antiplatelet therapy, at the cost of a higher rate of atrial fibrillation. In the CLOSE trial, for example, no recurrent strokes occurred in the closure group versus recurrences in the antiplatelet-only group, while atrial fibrillation was more frequent after closure^23,26^. Earlier trials were mixed or inconclusive, however, underscoring that net benefit depends heavily on patient selection and competing stroke mechanisms^23^. Closure is most beneficial in carefully selected patients, particularly those with high-risk anatomy, whereas medical therapy remains appropriate when the stroke mechanism is uncertain, closure is not feasible, or arrhythmia risk is a priority.

Migraine-focused cohorts suggest symptom improvement after closure in both device groups, but the evidence remains limited by non-randomised design and inconsistent reporting. Mechanistically, migraine improvement may relate to reduced passage of microemboli or vasoactive substances; however, randomised migraine trials have generally shown heterogeneous results, failing to meet some primary endpoints despite positive signals in secondary outcomes^15^. Nitinol devices, conversely, have been hypothesised to *contribute* to post-procedure symptoms through microthrombi or nickel hypersensitivity in susceptible patients^15,25,27^. On balance, closure may benefit some migraine patients, but certainty is low, and any advantage of biodegradable devices for symptom outcomes will require better long-term comparative evidence.

Biodegradable PFO occluders are designed to provide temporary scaffolding for closure, followed by gradual resorption—maintaining initial mechanical stability while reducing long-term intracardiac material. In the included studies, biodegradable devices used polymer-based components (e.g., PLA, PDO, or PLLA constructs) and showed progressive degradation on follow-up imaging^14,15^. Future development will likely focus on optimising the balance between degradation and endothelialisation (to limit residual shunt), improving mechanical properties for deployment, and extending long-term comparative follow-up to detect rare but clinically significant late events^14,24^.

### Strengths and limitations

This review has several strengths. It addressed a focused, clinically relevant comparative question; followed a prospectively registered protocol; applied duplicate study selection and data extraction; and used formal risk-of-bias tools for both randomised and observational evidence. It synthesised all currently available comparative studies of biodegradable versus nitinol PFO occluders—the only identified multicentre randomised trial alongside two comparative cohorts. Narrative synthesis was appropriate given the clear clinical and methodological heterogeneity across studies.

The review also has important limitations. The evidence base was small—three comparative studies with modest sample sizes—which limits precision and the ability to detect differences in uncommon adverse events. Two of the three were retrospective cohorts, and are therefore vulnerable to confounding, selection bias, and incomplete adjustment for baseline differences. Outcome reporting was heterogeneous, particularly for residual shunt and arrhythmia, varying in definitions, follow-up intervals, denominators, and surveillance methods, which reduced comparability across studies. Applicability was further limited because the evidence combined one mixed-indication RCT with two migraine-specific cohorts, reducing directness for stroke-prevention decision-making. All three studies were conducted in China, which may limit generalisability to other practice settings. Commercial concentration is a further concern: the biodegradable devices were linked to a narrow set of manufacturers—with explicit manufacturer involvement in Zhang et al. and manufacturer-specific devices or suppliers in Li et al. and Sun et al.—limiting independent replication. Two potentially relevant full-text reports could not be retrieved, raising the possibility of incomplete evidence capture. Finally, antithrombotic regimens differed across studies—dual antiplatelet therapy for 6 months in Zhang et al.; 6 months of dual therapy followed by single therapy to 12 months in Li et al.; and aspirin for 6 months with clopidogrel for 3 months in Sun et al.—which could influence both device-related thrombosis risk and the time course of shunt sealing, and represents an additional uncontrolled confounder in the two cohorts.

### Implications

Clinically, the available comparative evidence suggests that biodegradable occluders may be a potential alternative to conventional nitinol or metallic platforms in selected patients treated in experienced centres. The present evidence base is nonetheless too limited to support definitive conclusions about superiority, rare safety outcomes, or long-term comparative durability after biodegradation. Device selection should therefore remain individualised, guided by the clinical indication for closure, PFO anatomy, shunt severity, operator familiarity with the platform, and the capacity to provide structured follow-up imaging and rhythm surveillance. For research, future work should prioritise adequately powered multicentre randomised trials with standardised residual-shunt definitions, harmonised imaging protocols, systematic arrhythmia monitoring, transparent reporting of manufacturer involvement, and follow-up extending beyond the biodegradation period.

## Conclusions

On the currently available comparative evidence, biodegradable or bioresorbable PFO occluders appear to perform similarly to conventional nitinol or metallic devices over the short to mid term. Procedural success was consistently high across device types, and residual shunt generally declined over time irrespective of device material. However, variability in residual-shunt definitions (e.g., “any” versus “moderate-to-large”), differences in imaging protocols, and incomplete or inconsistent follow-up limit direct comparability and preclude firm conclusions about relative effectiveness. On safety, serious device-related complications, thrombosis, embolisation or detachment, and clinically detected arrhythmias were uncommon in both groups during the reported follow-up. Low event rates, heterogeneous surveillance, and limited arrhythmia monitoring nonetheless reduce confidence in detecting rare or transient events, so clinically meaningful differences cannot be excluded. Critically, no study followed patients beyond the biodegradation period, so the long-term advantage that motivates biodegradable platforms remains unproven, and the evidence base is drawn predominantly from migraine populations rather than the stroke-prevention indication for which PFO closure is established. The current evidence therefore does not demonstrate a clear advantage of biodegradable over nitinol or metallic occluders for the outcomes assessed, and the absence of an observed difference should not be interpreted as equivalence. Given the small evidence base, the predominance of retrospective data, and methodological heterogeneity, well-powered multicentre randomised trials with standardised outcome definitions and long-term follow-up are warranted before broader clinical recommendations can be made.

## Contributions of authors

**Conceptualization:** Abdulbari Alsaeed, Mohammed Qintar, Ahmad R. Al-Qudimat

**Methodology:** Abdulbari Alsaeed, Raed M. Al-Zoubi, Ahmad R. Al-Qudimat

**Literature search:** Abdulbari Alsaeed, Rufaidah H. Alameen, Osama Mohamad

**Screening:** Abdulbari Alsaeed, Rufaidah H. Alameen, Osama Mohamad

**Data extraction:** Abdulbari Alsaeed, Rufaidah H. Alameen, Osama Mohamad

**Risk of bias assessment:** Abdulbari Alsaeed, Rufaidah H. Alameen, Osama Mohamad

**Writing:** Abdulbari Alsaeed, Mohammed Qintar, Ahmad R. Al-Qudimat, Raed M. Al-Zoubi, Rufaidah H. Alameen, Osama Mohamad

**Review & editing:** Abdulbari Alsaeed, Raed M. Al-Zoubi, Mohammed Qintar

## Declarations of interest

All authors declare that they have no known competing financial interests or personal relationships that could have appeared to influence the work reported in this paper

## Funding

This research received no specific grant from any funding agency in the public, commercial, or not-for-profit sectors
